# Association of neonatal and fetal malformations with polyhydramnios and oligohydramnios - introduction of a new “association factor”

**DOI:** 10.1186/s12884-025-07797-5

**Published:** 2025-07-03

**Authors:** Artur Beke, Aténé Simonyi

**Affiliations:** Department of Obstetrics and Gynecology, Semmelweis University, Budapest, Hungary

**Keywords:** Neonatal malformations, Fetal structural abnormalities, Ultrasound, Polyhydramnios, Oligohydramnios

## Abstract

**Background:**

Our aim was to investigate the association of different neonatal and fetal anatomical abnormalities with polyhydramnios or oligohydramnios during prenatal ultrasonography.

**Methods:**

In our study, we processed prenatal sonographic and postnatal neonatal clinical and pathological data from 2,622 fetuses with malformations over a 12-year period. We investigated the association of neonatal and fetal abnormalities with polyhydramnios or oligohydramnios. To characterize the prevalence of association between a given disorder and polyhydramnios or oligohydramnios, we used our proprietary “association factor” (AF) for statistical calculations.

**Results:**

Amniotic fluid volume abnormalities were most frequently detected in urogenital, abdominal, and abdominal wall anomalies. In urogenital anomalies, amniotic fluid volume abnormalities were found in more than half of the fetuses, 54.86% (oligohydramnios 34.72%, polyhydramnios 20.14%). In abdominal and abdominal wall anomalies, 43.82% of fetuses had abnormalities in the volume of amniotic fluid (polyhydramnios 31.44%, oligohydramnios 12.38%). Overall, we found abnormalities in the volume of amniotic fluid in over 30% of the fetuses with craniospinal, thoracic and pulmonary anomalies, limb anomalies, and ossification disorders. In craniofacial and cardiovascular anomalies, amniotic fluid volume abnormalities were only detectable in around 20%. For polyhydramnios, the “Association Factor” (AF) was very high for craniospinal, abdominal, and abdominal wall disorders, and it was high for cardiovascular, urogenital, limb, and ossification disorders. For oligohydramnios, the association factor (AF) was very high for urogenital disorders.

**Conclusions:**

If the amniotic fluid volume during ultrasonography is less or more than average, special attention should be paid to the ultrasound examination of the fetus’ urogenital system, abdominal organs, skull, spine, chest, lungs, as well as limbs and skeletal system. In the case of polyhydramnios, fetal echocardiography is recommended.

## Background

To date, ultrasound screening and targeted ultrasound examinations have played the most important role in the detection of fetal malformations. Ultrasound is the most widely used medical imaging procedure in the world, it is extremely safe, and with the development of ultrasound systems, it has been associated with increasing imaging performance [1]. According to the Society for Maternal-Fetal Medicine, polyhydramnios and oligohydramnios are the two most commonly detected ultrasound abnormalities during pregnancy [2].

The possible causes of poly- and oligohydramnios are discussed in several publications [3–7]. Polyhydramnios and oligohydramnios may be associated with fetal anatomical abnormalities [8–12], fetal chromosomal abnormalities [13]. In a study spanning four years, Hentemann et al. investigated 140 pregnancies associated with various developmental abnormalities detected by ultrasound, the authors highlighted the importance of karyotyping from amniotic fluid in cases of detected developmental abnormalities and oligohydramnios [14]. Kouamé et al. point out that when polyhydramnios is detected, particular care must be taken during ultrasound examination in order to detect any fetal abnormalities that may be present [15].

The prevalence of fetal malformations at birth is around 2–3% [16–18]. Overall, polyhydramnios occurs in 1–2% of pregnancies, but the causes of polyhydramnios remain unknown in the majority of cases [6]. Polyhydramnios may be due to a congenital abnormality in 20% of cases, but in 60–70% of cases it is idiopathic, i.e., there is no identified cause of the increased amount of amniotic fluid [19].

Among the fetal malformations, amniotic fluid volume abnormalities may be associated with craniospinal [20, 21], craniofacial [8], cardiac developmental [22], and other thoracic abnormalities [23–25], abdominal and abdominal wall disorders [26–30], urogenital disorders [31–34].

In the case of less than average amniotic fluid, oligohydramnios, the amount of amniotic fluid is below normal (less than 500 ml in the second and third trimesters). The possible causes of oligohydramnios are various and include: fetal abnormalities, premature rupture of the membranes, placental insufficiency, deteriorating fetal circulation, insufficient maternal fluid intake, starvation and smoking. More than average amniotic fluid, polyhydramnios, is considered to be the case when there is a larger amount of amniotic fluid. Between 26 and 38 weeks, the volume of amniotic fluid exceeds 1500 ml and is still above 1000 ml after the 38th week [35]. It may be caused by an underlying maternal disease, a pregnancy-related condition (diabetes mellitus, diabetes gestationis), infection, fetal developmental abnormalities causing overproduction of amniotic fluid or impeding absorption or circulation, fetal circulatory abnormalities, twin pregnancies. The co-occurrence of fetal retardation and polyhydramnios may indicate chromosomal abnormalities and therefore cytogenetic testing is recommended [36, 37]. Most cases of mild polyhydramnios are idiopathic, and the two most common pathological causes of polyhydramnios include maternal diabetes mellitus and various fetal anomalies that may be associated with genetic syndromes [2]. The role of maternal diabetes mellitus or gestational diabetes in the development of polyhydramnios has been highlighted also by other authors [8]. When polyhydramnios is associated with fetal macrosomia, the most common maternal etiology is inadequately controlled and managed diabetes mellitus. Untreated maternal diabetes mellitus puts the fetus at lifelong risk of obesity and metabolic syndrome, and will have a higher rate of caesarean Sects. [38, 39].

Several objective methods can be used to characterise amniotic fluid volume. One method is to determine the vertical diameter of the single deepest pocket (SDP) of amniotic fluid. The normal value is 2–8 cm. In the case of polyhydramnios, the limit for the maximum vertical diameter of the amniotic fluid pocket is ≥ 8 cm [11, 40]. A mild case is defined as a maximum vertical diameter of 8–11 cm, a moderate case 12–15 cm, and a severe case of amniotic fluid gain of more than 16 cm. In the case of oligohydramnios, the limit for the maximum vertical diameter of the amniotic fluid pocket is < 2 cm [11]. This method is more commonly used in twin pregnancies [37].

The other method to measure amniotic fluid volume is the amniotic fluid index (AFI) measured by the four quadrant method. To calculate this, the maximum vertical thickness of the amniotic fluid in the four quadrants of the uterus, measured in centimeters, is added together. The normal value is 5–24 cm. Some authors have proposed ≥ 24 cm as the cut-off value for polyhydramnios [41, 42], others ≥ 25 cm, the latter being currently accepted [8, 13, 40]. Polyhydramnios can be defined as moderate if the amniotic fluid index is between 25 and 29.9 cm, intermediate if it is between 30 and 34.9 cm, and severe if it is above 35 cm [8]. For amniotic fluid index measured by the four quadrant method, the cut-off value for oligohydramnios is ≤ 5 cm [43].

**Fig. 1:**
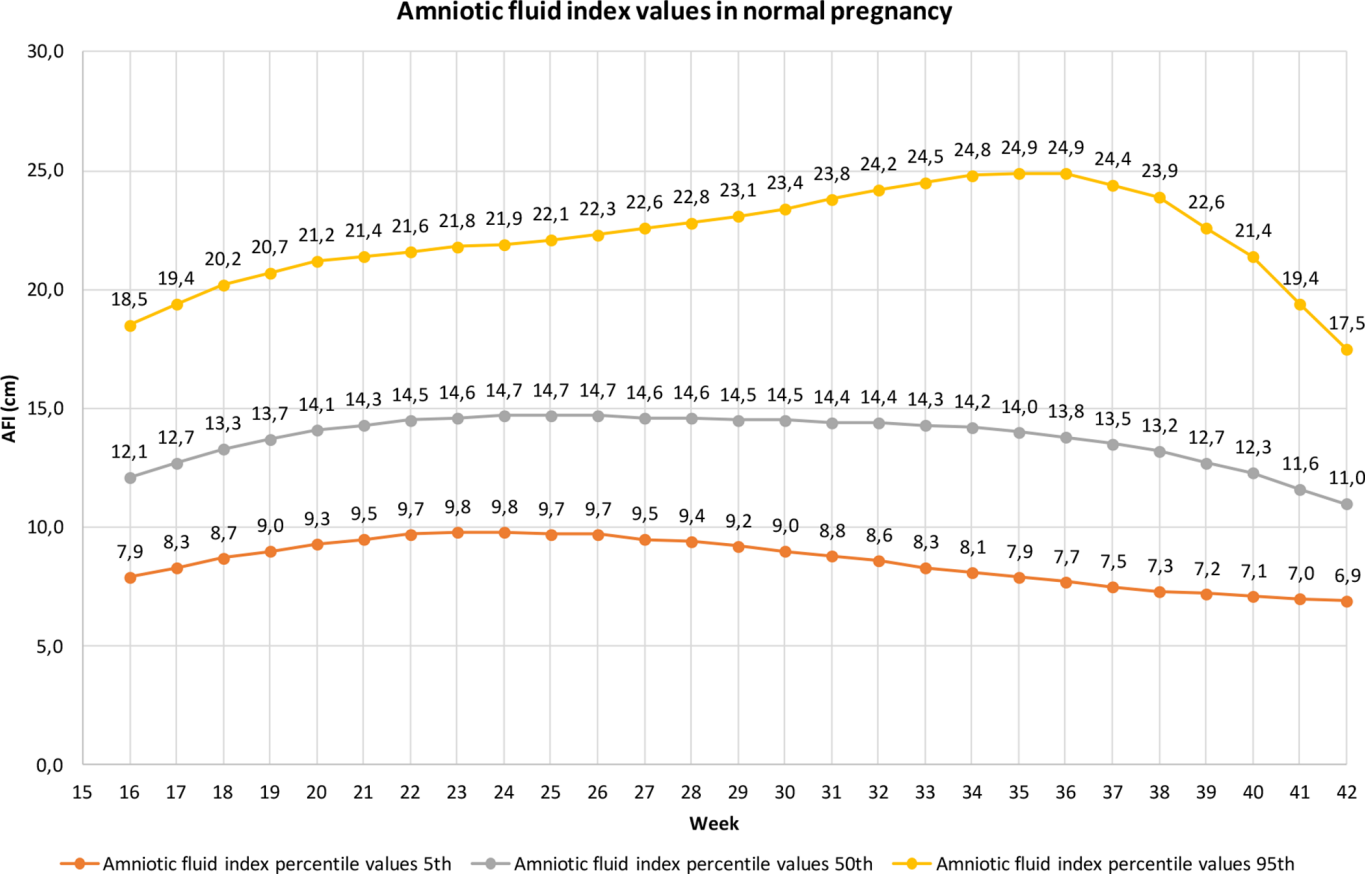
Average volume of amniotic fluid, AFI values in cm at the 5th percentile (oligohydramnios) and the 95th percentile (polyhydramnios) by week of gestation (own figure based on data from Moore and Cayle)

Moore and Cayle determined the value of the amniotic fluid index (AFI) per week of gestation (Fig. 1). They measured the values at the 2.5, 5, 50, 95, and 97.5 percentiles for each week of gestation [44]. Breaking down the values into weeks of gestation allows more accurate measurements. In the study reported in this article, this method was also used, and it is used to describe oligohydramnios below the 5 percentile and polyhydramnios above the 95 percentile (Fig. 1).

## Methods

In our study, we analyzed 12 years of data (2005–2016) from the I. Department of Obstetrics and Gynaecology, University of Semmelweis University, where developmental anomalies were diagnosed in the fetopathological findings or during postnatal examination. We processed prenatal ultrasound findings, postnatal clinical data, and fetopathological findings. We investigated the proportion of each fetal anatomical abnormality associated with polyhydramnios or oligohydramnios on prenatal fetal ultrasound screenings.

From 2005 we introduced in our department the method of amniotic fluid index (AFI) for measuring polyhydramnios and oligohydramnios. During the period of 2005–2016 in almost all cases the amniotic fluid index was used. From 2017 to 2018 in our department the single deepest pocket (SDP) was used. In the study, we aimed to apply a uniform method, which is why we decided to examine the period 2005–2016.

Polyhydramnios and oligohydramnios were defined in almost all cases using amniotic fluid index (AFI) values measured during ultrasound examinations and categorized as oligohydramnios at the 5th percentile and polyhydramnios at the 95th percentile. In only few cases, if no AFI value was available, the value of the single deepest pocket (SDP) was used.

For comparability with literature data, abnormalities were grouped according to the criteria of the EUROCAT study. The abnormalities were classified into major groups: craniospinal, craniofacial, cardiovascular, other thoracic abnormalities, abdominal and abdominal wall abnormalities, urogenital abnormalities, limb abnormalities, and ossification disorders. We also examined disorders associated with subcutaneous oedema.

Prenatally we used Fenton growth chart for diagnosis of fetal growth restriction (FGR). The estimated weight below 10th percentile was categorised as FGR.

The ultrasound examinations were performed in the Ultrasound Laboratory of the Clinic using Medison Sonoace X8 (Medison Co., LTD, Samsung Medison UGEO H60 (Samsung Medison Co., LTD), Samsung Medison WS80A (Samsung Medison Co., LTD), Philips^®^ HD 11XE (Philips Ultrasound) ultrasound machines.

To characterize the co-occurrence of polyhydramnios and oligohydramnios with fetal organ system abnormalities, we first used a linkage analysis. The Yule’s coefficient was used:

$$\text { Yule's coefficient: } \text Y=\frac{(A B \cdot \alpha \beta)-(A \beta \cdot \alpha B)}{(A B \cdot \alpha \beta)+(A \beta \cdot \alpha B)}$$

Where:

- AB: Fetal malformation + polyhydramnios/oligohydramnios.
- Aβ: Fetal malformation without polyhydramnios/oligohydramnios.
- αB: Polyhydramnios/oligohydramnios without fetal malformation.
- αβ: Cases without polyhydramnios/oligohydramnios and fetal malformation.

For evaluation:

• −1 < Y < 1

- ° Y = 0 - independent.
- ° Y = 1 - full definition.
- ° Y < 0.3 - weak association.
- ° 0.3 ≤ Y ≤ 0.7 - medium association.
- ° Y > 0.7 - strong association.

In order to find a more accurate and sensitive characteristic than the Yule’s coefficient, which better describes the association between a given disorder and polyhydramnios or oligohydramnios, we developed a new statistical parameter, the “Association Factor” (AF). In the following, we used the new “Association Factor” (AF) to characterize the intersection (F ∩ P or F ∩ O) of the sets F and P or O (where F: fetal malformations, P: polyhydramnios, O: oligohydramnios) in the statistical calculations.

**Fig. 2:**
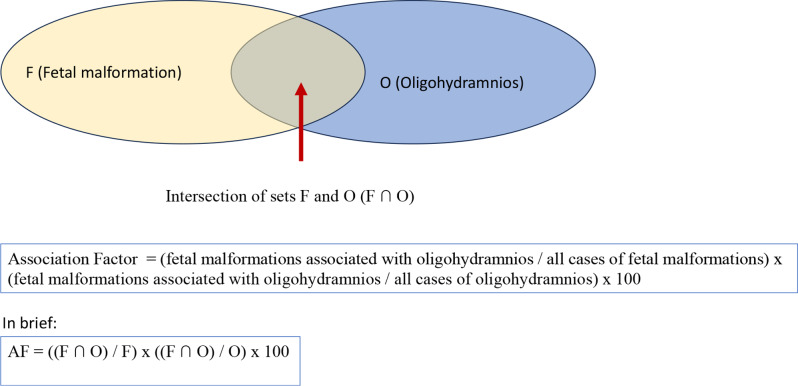
Calculation of association factor for oligohydramnios

Calculation of association factor for oligohydramnios (Fig. 2):

AF = (fetal malformations associated with oligohydramnios / all cases of fetal malformations) x (fetal malformations associated with oligohydramnios / all cases of oligohydramnios) x 100.

**Fig. 3:**
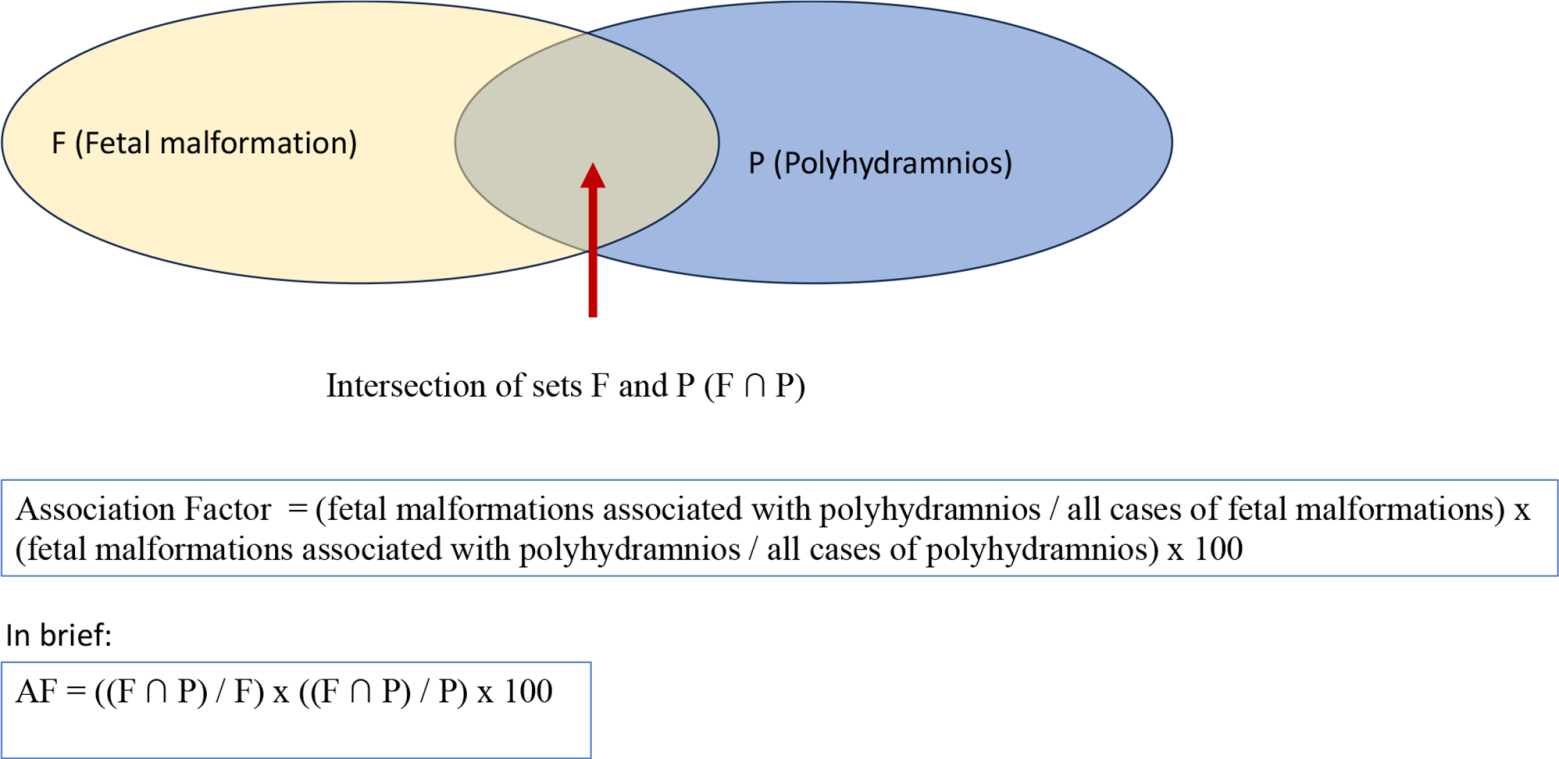
Calculation of association factor for polyhydramnios

Calculation of association factor for oligohydramnios (Fig. 3):

AF = (fetal malformations associated with oligohydramnios / all cases of fetal malformations) x (fetal malformations associated with oligohydramnios / all cases of oligohydramnios) x 100.

The following categories were defined for the assessment:

- AF < 0.5 - low.
- 0.5 ≤ AF < 2.50 - moderate.
- 2.5 ≤ AF < 5 -high.
- AF ≥ 5 - very high.

For statistical tests, the Chi-square test and Fisher’s Exact Test were used to calculate significance. An anomaly was considered statistically significant at *P* < 0.05P < 0.05. Our research conforms to the principles of the Declaration of Helsinki and was approved by the Ethics Committee of the Institution (Scientific Research Ethics Committee, approval number SE-TUKEB 231).

## Results

In 12 years, 41,069 deliveries and 1,957 mid-term abortion were recorded at the I. Department of Obstetrics and Gynaecology. Of the 1,957 mid-term abortions 1,440 were induced (TOP-termination of pregnancy), and 517 were spontaneous miscarriages. A total of 2,825 fetuses had some form of malformation during the observed period. Of the 2,825 cases, 203 cases were excluded from the study. Cases where no ultrasound examination was performed and the abnormality was detected only after delivery/miscarriage were excluded.

In total, data from 2,622 fetuses were processed, of which 1,256 resulted in a delivery. Of the 1,256 newborns born with malformations, 644 were born before 37 weeks of gestation, with a premature birth rate of 51.3%. Of the 2,622 cases studied, 1,366 were mid-term abortions, and post-miscarriage fetopathological examination was performed. Sixty-seven cases were spontaneous miscarriages, and in case of 1,299 fetuses, TOP was performed.

Craniospinal abnormalities occurred in 530 fetuses, and facial and neck malformations in 218 cases. The highest number of cases was cardiac and large vessel anomalies (818 cases). 156 cases of other thoracic anomalies were observed, 404 cases were included in the abdominal and abdominal wall anomalies, 432 cases in the urogenital anomalies group, while 255 cases were classified in the limb and ossification anomalies group. Abnormalities with subcutaneous oedema occurred in 243 fetuses (Table 1).

**Table 1:** Cumulative efficacy of fetal malformation detection for all fetuses

		Totally discovered		Not detected	
	cases	*n*	%	*n*	%
Craniospinal malformations	530	350	66.04%	180	33.96%
Craniofacial malformations	218	71	32.57%	147	67.43%
Cardiovascular malformations	818	508	62.10%	310	37.90%
Thoracic and pulmonary malformations	156	88	56.41%	68	43.59%
Abdominal and abdominal wall malformations	404	246	60.89%	158	39.11%
Urogenital malformations	432	224	51.85%	208	48.15%
Bone and limb developmental malformations	255	138	54.12%	117	45.88%
Malformations with subcutaneous oedema	243	191	78.60%	52	21.40%

Not all abnormalities are detected during pregnancy, and in some cases, the developmental abnormality is only detected after birth or during a fetopathological examination following a miscarriage. Over 50% of anatomical abnormalities in intrauterine fetuses were detected in craniospinal, cardiovascular, thoracic, abdominal, and abdominal wall anomalies, as well as in cases of subcutaneous oedema (Table 1).

Fetal structural abnormalities may be associated with chromosomal abnormalities and multiplex anomalies. The association of fetal chromosomal abnormalities can be seen in Table 2. 10% or more of chromosomal abnormalities were detected in craniofacial (33/218, 15.1%), cardiovascular (112/818, 13.7%), and limb anomalies and ossification disorders (26/255, 10.2%), and in abnormalities with subcutaneous oedema (42/243, 17.3%). Below 10% chromosomal abnormalities were found in craniospinal (43/530, 8.1%), thoracic (6/156, 3.8%), abdominal and abdominal wall (29/404, 7.2%), and urogenital (25/432, 5.8%) abnormalities.

**Table 2:** Summary table of the occurrence of chromosome abnormalities

	Number of fetuses		All chromosome abnormalities		Down (21 trisomy)	Edwards (18 trisomy)	Patau (13 trisomy)	Turner (X monosomy)	Klinefelter (47,XXY)	Other	rare autosomal trisomies (RAT)	translocation	autosomal deletion / duplication (CNV)	polyploidies	other autosomal chromosome abnormalities	other sex chromosome abnormalities
			*n*	%												
Craniospinal malformations	530		43	8.1%	17	17	2	1	0	6	2 × (15 trisomy, 46,XY / 47,XY + 20)	transloc (5;9)	13q del			2x (Xp del, X-ring)
Craniofacial malformations	218		33	15.1%	14	7	8	2	0	2	9 trisomy				22-ring	
Cardiovascular malformations	818		112	13.7%	63	35	5	0	0	9	4 × (9 trisomy, 20 trisomy, 15 trisomy, 48,XXY + 18)		13q del	2x triploidy	22-ring	X-ring
Thoracic and pulmonary malformations	156		6	3.8%	4	1	0	0	0	1			5p del			
Abdominal and abdominal wall malformations	404		29	7.2%	14	10	4	1	0	0						
Urogenital malformations	432		25	5.8%	4	9	6	2	0	4	2 × (17 trisomy, 9 trisomy)		13q del	triploidy		
Bone and limb developmental malformations	255		26	10.2%	8	10	3	0	0	5	2 × (9 trisomy, 48,XXY + 18)		2 × (1q del, 13q del)	triploidy		
Malformations with subcutaneous oedema	243		42	17.3%	19	6	0	15	0	2	2 × (17 trisomy, 9 trisomy)					

Multiple malformations are defined as those cases where two or more organ systems are affected, and no chromosomal abnormality is found in the underlying pathology. The following table shows that a high proportion of multiple malformations occurred in all organ systems. Craniospinal malformations accounted for 33%, craniofacial malformations for 49%, cardiovascular malformations for 28%, thoracic malformations for 67%, and abdominal and abdominal wall malformations for 39%. Additionally, 37% of urogenital disorders, 49% of limb disorders and ossification disorders, and 36% of disorders with subcutaneous oedema were associated with other organ system anomalies (Table 3).

**Table 3:** Summary table of incidence of multiple malformations

	All fetuses with malformation		Fetuses with multiple malformations		2 organ systems are involved	≥ 3 organ systems are affected	Craniospinal malformations	Craniofacial malformations	Cardiovascular malformations	Thoracic and pulmonary malformations	Abdominal and abdominal wall malformations	Urogenital malformations	Bone and limb developmental malformations	Malformations with subcutaneous oedema
			*n*	%										
Craniospinal malformations	530		174	33%	112	62		47	59	12	37	44	48	17
Craniofacial malformations	218		107	49%	57	50	47		38	13	20	25	33	8
Cardiovascular malformations	818		225	28%	137	88	59	38		39	75	73	35	16
Thoracic and pulmonary malformations	156		105	67%	49	56	12	13	39		37	26	19	36
Abdominal and abdominal wall malformations	404		158	39%	75	83	37	20	75	37		49	32	27
Urogenital malformations	432		158	37%	93	65	44	25	73	26	49		35	7
Bone and limb developmental malformations	255		124	49%	62	62	48	33	35	19	32	35		12
Malformations with subcutaneous oedema	243		87	36%	50	37	17	8	26	9	9	7	12	

In processing the cases, we investigated whether the diseased fetus was found to be in singleton or twin pregnancy. The percentage of twin pregnancies was over 10% for craniofacial anomalies, but it was also over 5% for cardiovascular anomalies, thoracic anomalies, abdominal and abdominal wall anomalies, as well as urogenital anomalies (Table 4).

**Table 4:** Summary table of fetuses with malformations from singular, gemini and trigeminal pregnancies

			Fetuses with malformations					
	All fetuses with malformation		Singular pregnancies		Gemini pregnancies		Trigeminal pregnancies	
			*n*	%	*n*	%	*n*	%
Craniospinal malformations	530		505	95.28%	23	4.34%	2	0.38%
Craniofacial malformations	218		193	88.53%	22	10.09%	3	1.38%
Cardiovascular malformations	818		756	92.42%	57	6.97%	5	0.61%
Thoracic and pulmonary malformations	156		143	91.67%	11	7.05%	2	1.28%
Abdominal and abdominal wall malformations	404		377	93.32%	25	6.19%	2	0.50%
Urogenital malformations	432		393	90.97%	38	8.80%	1	0.23%
Bone and limb developmental malformations	255		244	95.69%	11	4.31%	0	0.00%
Malformations with subcutaneous oedema	243		231	95.06%	12	4.94%	0	0.00%

### Yule’s association coefficient test

To characterize the co-occurrence of polyhydramnios and oligohydramnios with fetal organ system abnormalities, we first used an association test. Among international statistical methods, we used Yule’s coefficient to calculate the association coefficient.

In the calculation of the Yule’s coefficient for polyhydramnios, we found a strong association (Y > 0.7) for craniospinal, abdominal and abdominal wall, and urogenital disorders, as well as for limb disorders and ossification disorders. We found a moderate association (0.3 ≤ Y ≤ 0.70) for craniofacial, cardiovascular, other thoracic, and subcutaneous oedema disorders (Table 5).

**Table 5:**
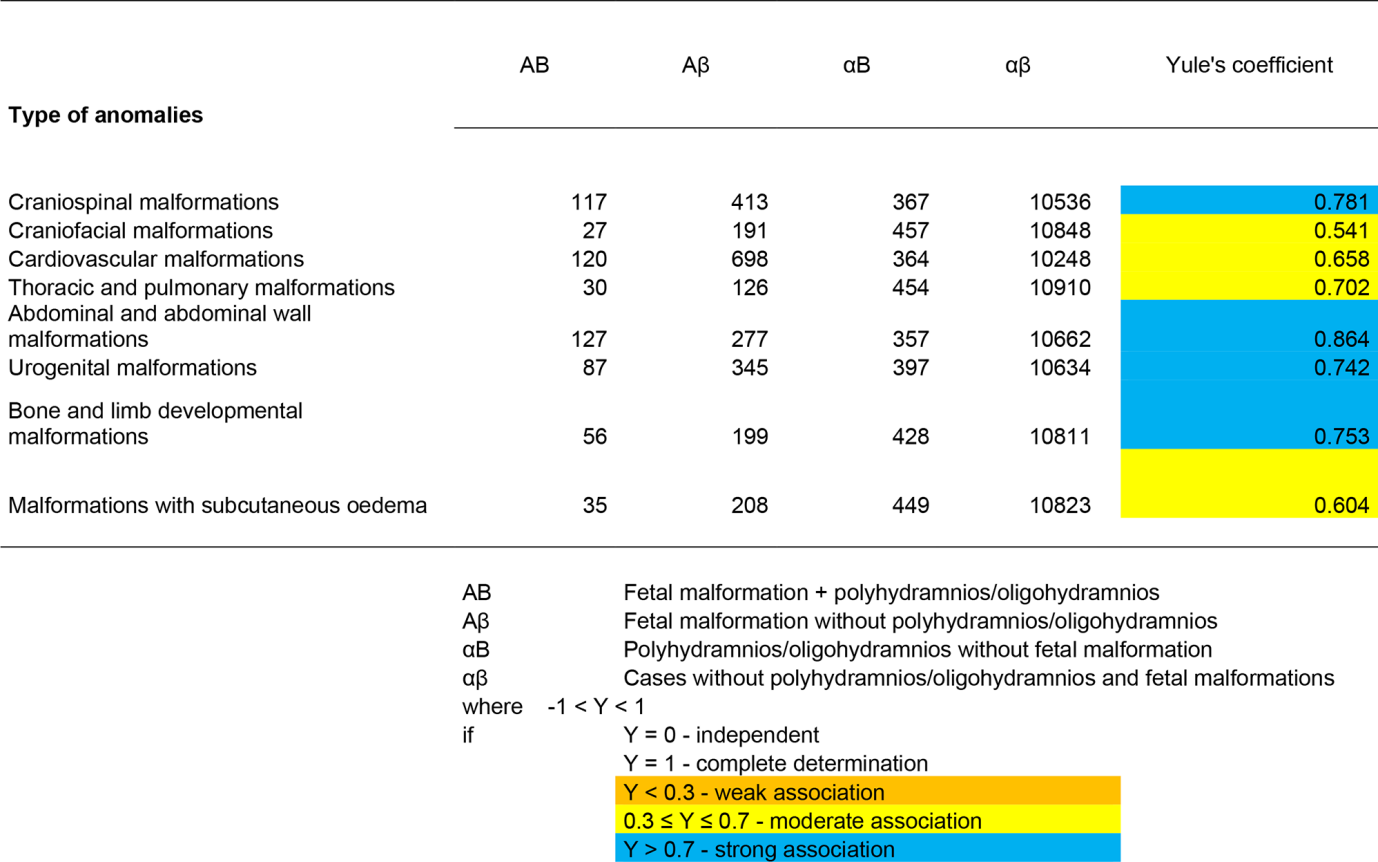
Calculation of association coefficient (Yule) in case of polyhydramnios

When calculating the Yule’s coefficient for oligohydramnios, we found a strong association (Y > 0.7Y > 0.7) for thoracic, abdominal and abdominal wall, and urogenital disorders. Whereas, we found a moderate association (0.3 ≤ Y ≤ 0.70) for craniospinal, craniofacial, cardiovascular, and limb and ossification disorders (Table 6).

**Table 6:**
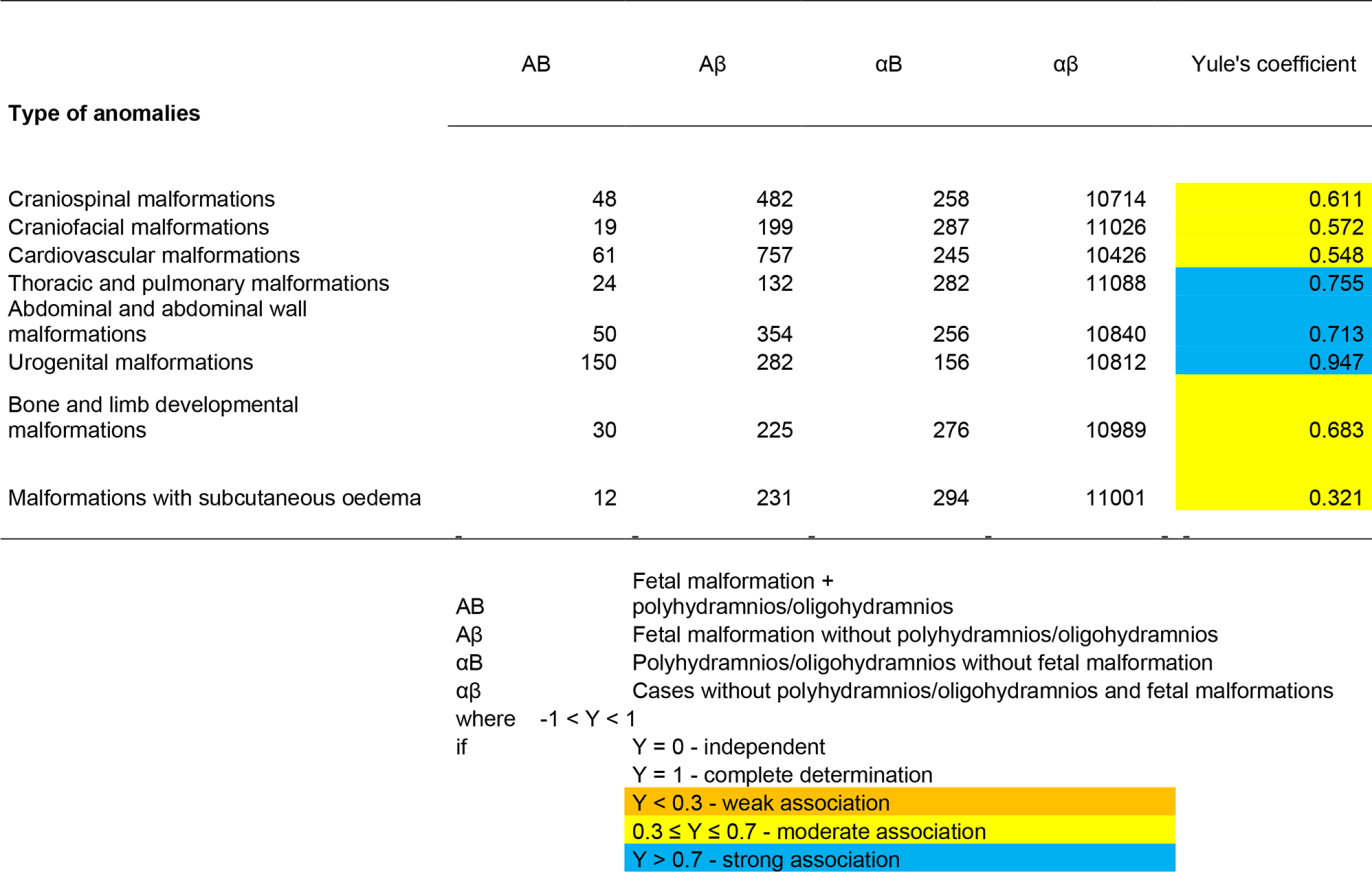
Calculation of association coefficient (Yule) in case of oligohydramnios

Considering that homogeneously moderate to strong associations were detected for both polyhydramnios and oligohydramnios, a new and better differentiating statistical method was developed.

### Association factor (AF) analysis

In order to find a more precise and sensitive characteristic than the Yule’s coefficient to describe more accurately the association between a given disorder and either polyhydramnios or oligohydramnios, we developed a new statistical parameter, the “Association Factor” (AF). The following values were used to calculate the AF: AF < 0.5: low, 0.5 ≤ AF < 2.5: moderate, 2.5 ≤ AF < 5: high, AF ≥ 5: very high.

In Table 7, we summarized the rates of abnormalities, fetal growth restriction, polyhydramnios and oligohydramnios per organ system. Significance was calculated to compare the ratio of polyhydramnios and oligohydramnios.

**Table 7:**
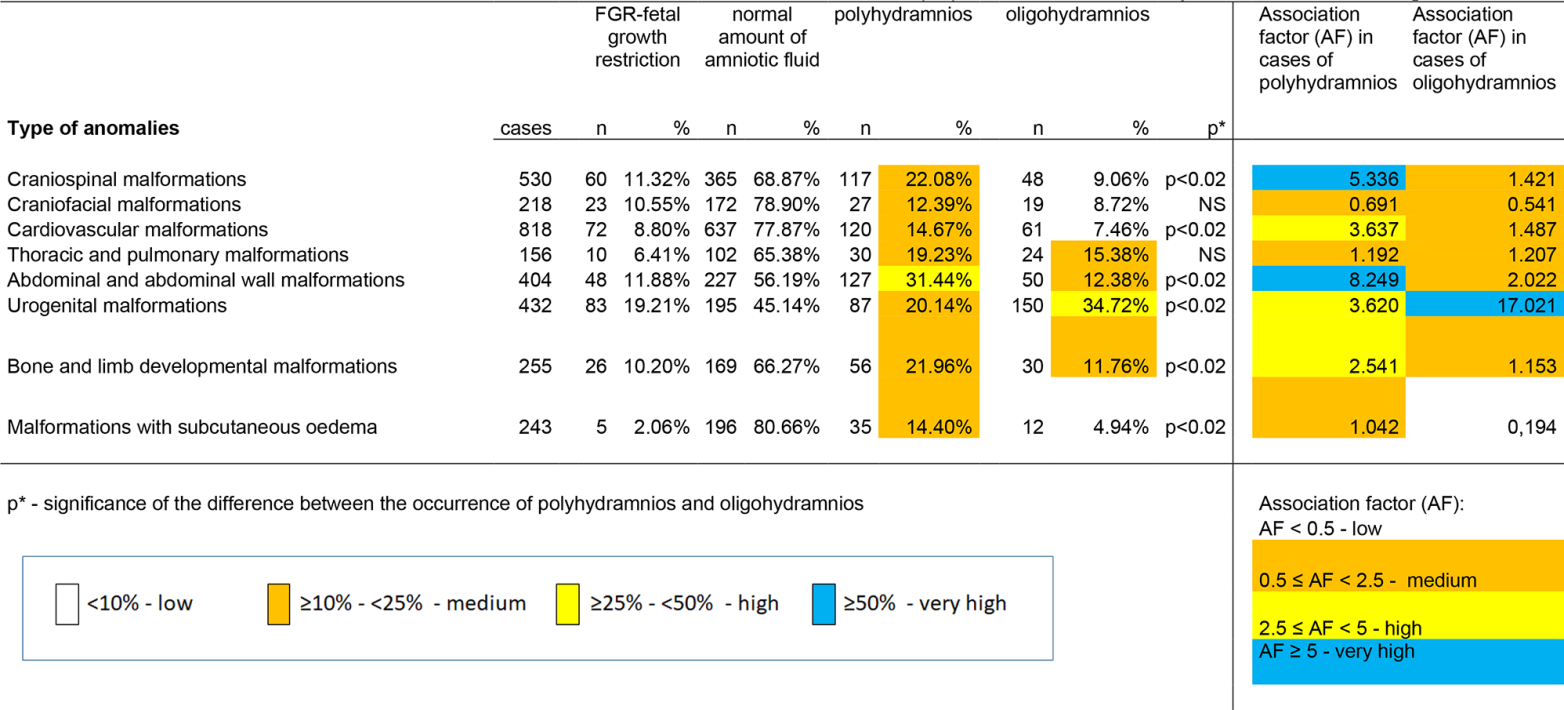
The rate of polyhydramnios, oligohydramnios, growth restriction and association factor (AF) in cases of fetal developmental malformations

A significantly higher incidence of amniotic fluid abnormalities was observed in cases involving craniospinal, cardiovascular, abdominal and abdominal wall disorders, as well as limb and ossification disorders. In contrast, there was a significantly higher incidence of oligohydramnios (34.72%) than polyhydramnios (20.14%) in urogenital disorders.

Our association factor (AF) for polyhydramnios showed a very high value for craniospinal, abdominal and abdominal wall anomalies (AF ≥ 5). The association factor was high for cardiovascular disorders, urogenital disorders, limb disorders, and ossification disorders (2.5 ≤ AF < 5), while the association factor was moderate for craniofacial, thoracic, and fetal disorders with subcutaneous oedema.

For oligohydramnios, the association factor was very high for urogenital disorders (AF ≥ 5) and moderate for craniospinal, craniofacial, cardiovascular, other thoracic, abdominal and abdominal wall disorders, as well as limb and ossification disorders (0.5 ≤ AF < 2.50).

### Association between intrauterine detectability of fetal abnormalities and amniotic fluid volume

Changes in amniotic fluid volume have been shown to affect the detectability of fetal anatomical abnormalities. Ultrasound screenings are also affected by the difficulty of the examination.

In the case of polyhydramnios, the fetus, and thus the fetal anatomical abnormalities, are easier to visualize during ultrasound examination, whereas in the case of oligohydramnios, ultrasound examination is more difficult. This explains why we have seen an upward trend in the detection of anatomical abnormalities in all organ groups in the case of polyhydramnios. Thus, a higher proportion of fetal anatomical abnormalities were detected with greater efficiency when associated with polyhydramnios. The difference was significant for craniofacial abnormalities, cardiovascular abnormalities, and thoracic abnormalities, with an increase in the rate of in utero detection from 32.57 to 51.85% for craniofacial abnormalities, from 62.1 to 79.69% for cardiovascular abnormalities, and from 56.41 to 74.19% for thoracic abnormalities (Table 8.).

**Table 8:** Recognition of fetal malformations associated with polyhydramnios

	All malformations			Associated with polyhydramnios				
		recognized			recognized		tendency	p
	cases	n	%	cases	n	%		
Craniospinal malformations	530	350	66.04%	117	94	80.34%	increased	NS
Craniofacial malformations	218	71	32.57%	27	14	51.85%	increased	*p* < 0.02
Cardiovascular malformations	818	508	62.10%	128	102	79.69%	increased	*p* < 0.05
Thoracic and pulmonary malformations	156	88	56.41%	31	23	74.19%	increased	*p* < 0.05
Abdominal and abdominal wall malformations	404	246	60.89%	129	82	63.57%	increased	NS
Urogenital malformations	432	224	51.85%	87	51	58.62%	increased	NS
Bone and limb developmental malformations	255	138	54.12%	61	36	59.02%	increased	NS
Malformations with subcutaneous oedema	243	191	78.60%	35	32	91.43%	increased	NS

When the fetal anatomical anomaly was associated with oligohydramnios, only cardiovascular, urogenital, limb anomalies, and ossification disorders, as well as anomalies with subcutaneous oedema, showed an upward trend in the detection of anomalies. However, in none of these cases was this trend significant.

For other organ systems, a decreasing trend in detectability was observed, and this trend was significant for craniospinal, craniofacial, thoracic, abdominal, and abdominal wall anomalies. In craniospinal anomalies, the intrauterine detection rate decreased from 66.04 to 39.58%, while in craniofacial anomalies, it decreased from 32.57 to 15.79%. In thoracic anomalies, it decreased from 56.41 to 41.67%, and in abdominal and abdominal wall defects, it decreased from 60.89 to 40.38% (Table 9).

**Table 9:** Recognition of fetal malformations associated with oligohydramnios

	All malformations			Associated with oligohydramnios				
		recognized			recognized		tendency	p
	cases	n	%	cases	n	%		
Craniospinal malformations	530	350	66.04%	48	19	39.58%	decreased	*p* < 0.02
Craniofacial malformations	218	71	32.57%	19	3	15.79%	decreased	*p* < 0.02
Cardiovascular malformations	818	508	62.10%	67	44	65.67%	increased	NS
Thoracic and pulmonary malformations	156	88	56.41%	24	10	41.67%	decreased	*p* < 0.05
Abdominal and abdominal wall malformations	404	246	60.89%	52	21	40.38%	decreased	*p* < 0.02
Urogenital malformations	432	224	51.85%	151	85	56.29%	increased	NS
Bone and limb developmental malformations	255	138	54.12%	45	27	60.00%	increased	NS
Malformations with subcutaneous oedema	243	191	78.60%	12	10	83.33%	increased	NS

### Prevalence of fetal growth restriction by organ system

In this study, we investigated the prevalence of fetal growth restriction (FGR), when the estimated intrauterine weightof a fetus is less than 10th percentiles of gestational age. Our results showed a higher rate of fetal growth restriction in urogenital anomalies, in almost 20% of fetuses (83/432 cases, 19.21%). In some of the organ systems, we found rates of around 10%: craniospinal anomalies (60/530, 11.32%), craniofacial anomalies (23/218, 10.55%), abdominal and abdominal wall defects (48/404, 11.88%), limb anomalies, and ossification disorders (26/255, 10.20%). Some organ systems showed lower values, below 10%: cardiovascular disorders (72/818, 8.80%), thoracic disorders (10/156, 6.41%), and disorders with subcutaneous oedema (5/243, 2.06%).

## Discussion

In our study data from 2,622 fetuses with structural malformations were processed, of which 1,256 resulted in a delivery. Amniotic fluid volume abnormalities were most frequently detected in urogenital, abdominal and abdominal wall anomalies. In urogenital anomalies, oligohydramnios occurred in most cases, 34.72%, while polyhydramnios occurred in 20.14%. In abdominal and abdominal wall anomalies, polyhydramnios occurred in most cases, 31.44%, while oligohydramnios occurred in 12.38%. In craniospinal anomalies, polyhydramnios was detected in most cases, 22.08%, and oligohydramnios in 9.06%. In thoracic and pulmonary anomalies, polyhydramnios occurred in 19.23%, and oligohydramnios occurred in 15.38%. In the case of limb anomalies and ossification disorders, polyhydramnios occurred in 21.96% and oligohydramnios in 11.76%. In the case of craniofacial anomalies, polyhydramnios occurred in 12.39% of fetuses and oligohydramnios in 8.72% during pregnancy. In the case of cardiovascular anomalies, polyhydramnios occurred in 14.67% and oligohydramnios in 7.46%.

Regarding polyhydramnios, the Association Factor (AF) we developed showed a very high value for craniospinal and abdominal and abdominal wall abnormalities (AF ≥ 5), high AF for cardiovascular abnormalities, urogenital abnormalities, limb abnormalities and ossification disorders (2.5 ≤ AF < 5), while AF was moderate for craniofacial, thoracic and fetal abnormalities with subcutaneous oedema. Regarding oligohydramnios, AF showed a very high value for urogenital abnormalities (AF ≥ 5). The value was moderate for craniospinal, craniofacial, cardiovascular, other thoracic, abdominal and abdominal wall abnormalities, and limb abnormalities and ossification disorders (0.5 ≤ AF < 2.5).

Polyhydramnios and oligohydramnios can have an impact on perinatal outcome [40]. Aviram et al. reported that, comparing normal and gravid women with AFI greater than 20 cm after 34 weeks of gestation, cases with polyhydramnios had a higher rate of placental abruption and abnormal fetal heart rate [45]. Compared to normal pregnancies, polyhydramnios may be associated with higher rates of postpartum hemorrhage, cesarean section, and neonatal respiratory distress [46]. In addition to the increased justification for cesarean section and the risk of placental abruption, other authors have also highlighted the importance of increased observation, as polyhydramnios may lead to more frequent intrauterine death and prolonged dilation (first stage of labour) [47]. Other authors point to more frequent postpartum respiratory morbidity in polyhydramnios [48]. In association with polyhydramnios, Walter et al. measured fetal growth restriction (FGR) in 4.6% of fetuses [49]. Burwick et al. studied cases of fetal hydrops and measured increased volume of amniotic fluid in 39.8%. Of the 337 cases of hydrops, an increased rate of preeclampsia, preterm delivery, and neonatal death was observed [50].

Isolated oligohydramnios are associated with an increased risk of placental abnormalities [51]. Zilberman et al. investigated the extent of oligohydramnios. Abnormally reduced amniotic fluid volume was associated with an increased rate of fetal cardiac arrhythmias, more frequent need for induction of labour, and worse neonatal outcome [52]. Oligohydramnios are also associated with more frequent cesarean section in low-risk pregnancies and an increased rate of low birth weight neonates [53]. Fetal weight estimation may also be confounded by lower than average amniotic fluid, and in oligohydramnios, birth weight may be overestimated even shortly before term [54]. If the height of the uterine fundus is lower than expected based on the calculated gestational age, reduced volume of amniotic fluid should be considered, and measurement is recommended [55].

Severe oligohydramnios can trigger pulmonary hypoplasia [56]. An international study found that children with reduced amniotic fluid volume had an 8% higher rate of clinical airway treatment and an 80% higher incidence of respiratory failure compared to children with no oligohydramnios detected [57]. Severe oligohydramnios can also have long-term neurological consequences for the newborn, which may be reflected in movement disorders [58].

### Association of fetal malformations with polyhydramnios and oligohydramnios

In the case of anatomical structural abnormalities of various organ systems, several publications have described that it is more often associated with polyhydramnios or oligohydramnios, but specific percentages are rarely included in the literature. In the case of craniospinal anomalies, exencephaly/anencephaly, which can be classified as neural tube defects based on literature data, is often associated with polyhydramnios [20]. In our own material, we found polyhydramnios in 22.08% of craniospinal anomalies. In the case of craniofacial anomalies, cleft lip and palate can lead to an increase in the amount of amniotic fluid due to difficulty swallowing [8]. In our own material, polyhydramnios occurred in 12.39% of all craniofacial anomalies. Cardiovascular disorders are often associated with polyhydramnios according to the literature [22]. In our own material, polyhydramnios occurred in 14.67% of cases of heart and great vessel disorders. In the case of thoracic and pulmonary disorders, congenital pulmonary airway malformation can lead to polyhydramnios if the lesion dislocates the mediastinal structures and causes compression of the oesophagus, thereby making swallowing and circulation of amniotic fluid difficult [10, 23, 24]. In our own material, polyhydramnios occurred in 19.23% of cases of thoracic and pulmonary disorders, and oligohydramnios occurred in 15.38%. Among abdominal and abdominal wall anomalies, according to the literature, in addition to oesophageal atresia, duodenal atresia and other small intestinal atresia are often associated with polyhydramnios [29]. Among abdominal wall anomalies, gastroschisis is often associated with polyhydramnios. In all abdominal and abdominal wall anomalies, polyhydramnios occurred in 31.44% of cases, and oligohydramnios occurred in 12.38%. Urogenital anomalies may be associated with polyhydramnios or oligohydramnios, and may affect the development of other organs [31, 33, 34]. In all urogenital anomalies, polyhydramnios occurred in 20.14% of cases, and oligohydramnios occurred in 34.72%. Among limb anomalies and ossification disorders, Almeida et al. reported an exceptionally high rate of polyhydramnios (38.9%) in skeletal system anomalies [59]. In our own material, polyhydramnios occurred in 21.95% of all limb anomalies and ossification disorders, and oligohydramnios in 11.76%.

Tsakmaki et al. in a meta-analysis included a total of 12 studies (2561 pregnancies complicated by isolated hydramnios). The prevalence of genomic anomalies in fetuses with apparently isolated polyhydramnios (12 studies, 2634 fetuses) was 4.5%. The pooled prevalence of chromosomal abnormalities (11 studies, 2427 fetuses) was 2.1% [60]. We carried out the studies from the opposite direction, in our research we did not examine the occurrence of malformations in the case of isolated polyhydramnios, but we examined the occurrence of polyhydramnios and oligohydramnios in the case of confirmed structural anomalies. We also examined the rate of chromosomal abnormalities. In addition to common aneuploidies, we also examined the occurrence of other rarer chromosomal abnormalities, e.g. rare autosomal trisomies (RAT), translocation, autosomal deletion / duplication (CNV), polyploidies. In cases of craniospinal malformations the rate of chromosomal anomalities was 8.1%, in cases of craniofacial malformations 15.1%, in cases of cardiovascular malformations 13.7%, in cases of abdominal and abdominal wall malformations 7.2%, in urogenital malformations 5.8% and in cases with bone and limb developmental malformations 10.2%, respectively.

In the calculation of the Yule’s coefficient for polyhydramnios, we found a strong association (Y > 0.7Y > 0.7) for craniospinal, abdominal and abdominal wall disorders, urogenital disorders, limb, and ossification disorders, and a moderate association (0.3 ≤ Y ≤ 0.70) for craniofacial, cardiovascular, other thoracic abnormalities, and disorders with subcutaneous oedema.

When calculating the Yule’s coefficient for oligohydramnios, we found a strong association (Y > 0.7Y > 0.7) for thoracic, abdominal and abdominal wall, and urogenital anomalies. Whereas, we found a moderate association (0.3 ≤ Y ≤ 0.7) for craniospinal, craniofacial, cardiovascular, and limb and ossification anomalies.

Considering that homogeneously moderate or strong associations (only two categories) were detected by classic statistical method, Yule’s coefficient for both polyhydramnios and oligohydramnios, a new and better differentiating statistical method was developed by us. In order to find a more precise and sensitive characteristic than the Yule’s coefficient to describe more accurately the association between a given disorder and either polyhydramnios or oligohydramnios, we developed a new statistical parameter, the “Association Factor” (AF).

The new method is allowing to describe by four categories e.g. AF was low (AF < 0.5 ) in cases of subcutneous oedema with oligohydramnios, AF was in medium range (0.5 ≤ AF < 2.5) in cases of craniofacial, thoracic, and subtutaneous oedema wit polyhydrmnios, and in cases of craniospinal, craniofacial, cardiovascular, thoracic, abdominal and bone malformations with oligohydramnios. The AF was high (2.5 ≤ AF < 5) in cases of cardiovascular, urogenital, and bone malformations with polyhydramnios, and AF was very high (AF ≥ 5) in caes of craniospinal and abdominal malformations with polyhydramnios, and in cases of urogenital anomalies with oligohydramnios.

### Association between intrauterine detectability of fetal abnormalities and amniotic fluid volume

Changes in amniotic fluid volume have been shown to affect the detectability of fetal anatomical abnormalities. Ultrasound screenings are also affected by the difficulty of the examination.

In the case of polyhydramnios, the fetus, and thus the fetal anatomical abnormalities, are easier to visualize during ultrasound examination, whereas in the case of oligohydramnios, ultrasound examination is more difficult. This explains why we have seen an upward trend in the detection of anatomical abnormalities in all organ groups in the case of polyhydramnios. Thus, a higher proportion of fetal anatomical abnormalities were detected with greater efficiency when associated with polyhydramnios. The difference was significant for craniofacial abnormalities, cardiovascular abnormalities, and thoracic abnormalities, with an increase in the rate of in utero detection from 32.57 to 51.85% for craniofacial abnormalities, from 62.1 to 79.69% for cardiovascular abnormalities, and from 56.41 to 74.19% for thoracic abnormalities.

Changes in amniotic fluid volume alone may also alert to an underlying fetal abnormality. It is often observed that the abnormality in amniotic fluid volume is the reason for sending the expectant mother for our genetic counselling, and that subsequent ultrasound scans and fetal echocardiography reveal additional fetal anatomical abnormalities.

Oligohydramnios, although it can raise awareness of fetal abnormalities, can also complicate ultrasound examination, which explains why we saw a non-significant upward trend in the detection of abnormalities only in cardiovascular, urogenital, limb and ossification disorders and disorders with subcutaneous oedema, and a downward trend in the detection of all other organ systems. This decreasing trend was significant in all cases, i.e. craniospinal, craniofacial, thoracic, abdominal and abdominal wall anomalies.

### Detection of fetal abnormalities and association with other abnormalities

For many organ systems, the detection rate was higher when the abnormality occurred alone. The results of the study raise the question of how to explain the lower detection rate when the anomaly is associated with a chromosomal abnormality or occurs as part of other multiplex abnormalities. It is important to consider that some of the abnormalities alone may justify the parents’ request for termination of the pregnancy. Therefore, in such cases, no further investigations are carried out, and other abnormalities may be detected during the fetopathological examination. It is also important to note that some of these abnormalities may not be detected until later in pregnancy or until after birth, and therefore may not be detected during intrauterine ultrasound scans in association with other abnormalities.

Strength of the study is that the new information primarily relates to the fact that we determined the exact rate of polyhydramnios or oligohydramnios in each organ system using the same method, and we determined the association between anatomical abnormalities and the amount of amniotic fluid using both the traditional and the new method we developed.The new method introduced, the determination of the “Association Factor” (AF), may also be suitable for performing other association statistical studies.

A limitation of the study is that the new method has not yet been tested to examine the association of other abnormalities; this is planned in a further publication.

## Conclusions

Our findings indicate that if the ultrasound examination reveals less or more amniotic fluid than the average, increased attention should be paid to ultrasound examination of the urogenital system, abdominal organs, skull and spine, and limbs and skeletal system of the intrauterine fetus. In the case of polyhydramnios, fetal echocardiography is recommended. The attention of the neonatologist or pediatrician should also be drawn to abnormalities in the volume of amniotic fluid at delivery, as further examination of the newborn may be warranted in these cases.

## Data Availability

The datasets used and/or analysed during the current study available from the corresponding author on reasonable request.
